# Morphological and antioxidant responses of *Nopalea cochenillifera* cv. Maya (edible *Opuntia* sp. “Kasugai Saboten”) to chilling acclimatization

**DOI:** 10.1007/s10265-023-01437-9

**Published:** 2023-01-23

**Authors:** Ayumu Kondo, Masashi Ito, Yusaku Takeda, Yuka Kurahashi, Shigeo Toh, Toru Funaguma

**Affiliations:** Faculty of Agriculture, Meijo University, 1–501 Shiogamaguchi, Tempaku, Nagoya, 468-8502 Japan

**Keywords:** Cactus, Chilling acclimatization, Chloroplast, Cuticular wax, Low temperature

## Abstract

To clarify the wintering ability of the cactus *Nopalea cochenillifera* cv. Maya (edible *Opuntia* sp., common name “Kasugai Saboten”), we investigated the effects of temperature and antioxidant capacity on chilling acclimatization. We analyzed the anatomy of cladode chlorenchyma tissue of plants exposed to light under chilling. We found that chilling acclimatization can be achieved by exposure to approximately 15 °C for 2 weeks and suggest that it is affected by whether or not antioxidant capacity can recover. The overwintering cacti had the thinnest cuticle but firm cuticular wax, which is important in the acquisition of low temperature tolerance under strong light. In cacti with severe chilling injury, round swollen nuclei with clumping chloroplasts were localized in the upper part (axial side) of the cell, as though pushed up by large vacuoles in the lower part. In overwintering cacti, chloroplasts were arranged on the lateral side of the cell as in control plants, but they formed pockets: invaginations with a thin layer of chloroplast stroma that surrounded mitochondria and peroxisomes. Specific cellular structural changes depended on the degree of chilling stress and provide useful insights linking chloroplast behavior and structural changes to the environmental stress response.

## Introduction

The cactus family (*Cactaceae*) contains approximately 1600 species in 100 genera. The plants of this family are native mainly to the Americas, with centers in Mexico and the southwestern USA (Barthlott and Hunt 1993). The species of *Opuntia* and *Nopalea* (subfamily *Opuntioideae*; *Opuntia* spp.) have been used as traditional food in Mexico; tender young cladodes used as vegetables are called *nopal*, and fruits are called *tuna* (Cushman et al. 2015; Yang et al. 2015). Young cladodes and fruits are also dried and sold as dietary supplements (Bensadon et al. 2010; Sáenz-Hernández et al. 2002), in cosmetic formulations (Sáenz-Hernández et al. 2002), and in medicine (de los Angeles Aguilera-Barreiro et al. 2013; Feugang et al. 2006; Stintzing and Carle 2005). In Brazil, these cacti are important forages in arid and semi-arid regions, especially the genotype Baiana of *Nopalea cochenillifera* (L.) Salm-Dyck, which has been shown to be most suitable for forage production even in different semi-arid tropical climates, such as Hot semi-arid and Tropical savanna (Edvan et al. 2020).

In Japan, cacti are mainly ornamental plants, but edible cacti are grown in greenhouses in some areas. Kasugai City, close to Nagoya, Aichi Prefecture, in the center of Japan, boasts the highest production of cacti in the country. Recently, Kasugai City has developed a “Kasugai Cactus Brand” project to promote regional development with cacti as a resource for regional vitalization. Processed food made of *Opuntia* spp. has been developed to expand the range of cactus use. Field culture is effective in increasing cactus production for food, but only empirical observations are available about how the cacti from tropical and subtropical regions grow in temperate regions such as those in Japan.

**Fig. 1:**
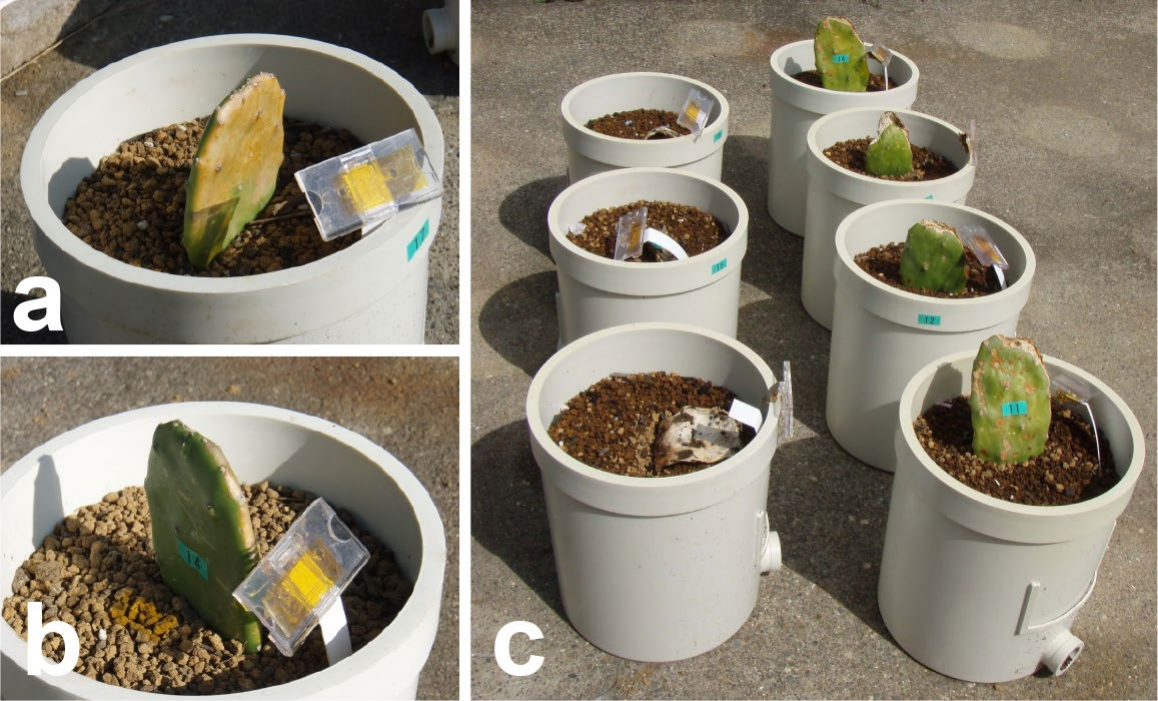
Effect of sunlight on *Nopalea cochenillifera* growth in winter (reproduced from Hosokawa et al. 2019). Plants were moved from indoors to outdoors on 1 December 2015. Photographs were taken on **a**, **b** 4 January and **c** 9 April 2016. At low temperature, the cladode with a large light-receiving surface (**a**) was severely injured by “leaf burn” and died (**c**, left-row), whereas the cladode with a small light-receiving surface (**b**) survived (**c**, right-row)

In our previous study, Kasugai Saboten (edible *N. cochenillifera* cv. Maya) in pots was grown outdoors instead of indoors, and the growth characteristics were investigated (Hosokawa et al. 2019). We revealed that it can grow outdoors even from December to October in Nagoya (average monthly air temperature: minimum 5.7 °C; maximum 30.1 °C); new cladodes grew at an average monthly air temperature of atleast 20 °C. However, in the cold season, the bigger the light-receiving side a plant had, the more it was damaged and likely to die (Fig. 1). The cacti are low temperature tolerant, but these observations suggest that intense light at low temperature induces cold damage. Cacti had insufficient time to adapt to the low temperature, because the treatment started in early December just after cacti grown in greenhouses were distributed (May–November), when the temperature was low (Hosokawa et al. 2019).

Chilling and freezing injuries are two main types of cold damage (Levitt 1980). The former is common in plants of tropical and subtropical origin such as legumes, maize, rice, and tomatoes, and is characterized by wilting and inhibition of growth, germination, and reproduction, and may cause complete tissue necrosis; it occurs at tissue temperatures below 8–10 °C in sensitive species. The latter is caused by partial freezing of water in tissues. Despite differences in sensitivity, all growing tissues are to some extent freeze-sensitive. In many non-acclimatized plants, freezing at − 1 to − 3 °C leads to tissue death.

Many cactus species originate in the arid and hot subtropical and tropical regions of the Americas, but 34% of species are native to regions in the USA and Canada where they are exposed to sub-freezing temperatures for at least several weeks each year (Loik and Nobel 1993). *Opuntia humifusa* has excellent freezing resistance; it starts to accumulate polysaccharides in cells around the onset of a cold winter, and increases osmotic pressure in preparation for dehydration stress caused by freezing (Loik and Nobel 1991). When the temperature decreases from 30 to 10 °C during the day and from 20 to 0 °C at night, osmolarity of cladode segments of *O. humifusa* is four times those of non-freeze-tolerant subtropical *Opuntia ficus-indica* and *Opuntia streptacantaba* (Goldstein and Nobel 1994).

It is not clear how freeze-tolerant the cactus in our study is, but the influence of temperatures of 5–10 °C is greater than the influence of those that below freezing (Hosokawa et al. 2019).

Photoirradiation of low-temperature-sensitive plants quickly causes chlorosis, which impairs photosynthetic ability and promotes cell damage (Jalil et al. 2017; Jung and Steffen 1997). In particular, damage to the photosynthetic electron transport chain promotes the production of active oxygen, and if the scavenger system does not function well at low temperatures, not only chloroplasts but also whole cells are fatally damaged (Coates et al. 2016). Such injury is called light inhibition. Thus, cold damage in cacti may contribute to light inhibition at low temperatures.

Oxidative stress induced by chilling may play a pivotal role in chilling injury in plant cells (Sachdev et al. 2021). It is mediated by reactive oxygen species (ROS), such as superoxide anion (O_2_^−^) and hydrogen peroxide (H_2_O_2_). Superoxide dismutase (SOD) catalyzes the disproportionation of superoxide to H_2_O_2_ and O_2_, and is important in protection against superoxide-derived oxidative stress in plant cells (Gill and Tuteja 2010).

In many plants, exposure to low temperatures for a certain period leads to a decrease in the temperature at which they are damaged by cold or freezing, a phenomenon called acclimatization to low temperature (Levitt 1980). Several biochemical and physiological changes in the antioxidant defense system are involved in chilling acclimatization, but little is known about cell ultrastructure under chilling conditions combined with light. We expected the intracellular structure to differ notably between wintering and non-wintering cacti, resulting in a difference in the degree of cold damage to the surface of the cladodes.

The purpose of this study was to investigate the ability of the cactus to winter under chilling conditions combined with light. We investigated the effects of temperature on SOD activity during chilling acclimatization and analyzed the anatomy of chlorenchyma tissue of the cladode side exposed to light under chilling conditions.

## Materials and methods

### Plant material and growth conditions

*Nopalea cochenillifera* cv. Maya (edible *Opuntia* sp., common name “Kasugai Saboten”; subfamily *Opuntioideae*, family *Cactaceae*) was bought at a local market (Goto Cactus, 1-122-3, Momoyama, Kasugai City, Aichi Prefecture, Japan). The cladode was dried for 10 days in the dark at room temperature and then placed in a 1 dm^−3^ pot filled with a mixture of field soil and leaf mold (7:3, v/v) for about 3 weeks until rooting in a glass greenhouse (Meijo University Tempaku Campus, Tempaku, Nagoya City, Aichi Prefecture, Japan). The greenhouse was warmed by boilers at ≥ 10 °C from November to March.

### Early, middle, and late treatments

Plants were either grown in glass greenhouses throughout the experiment (control) or moved outside at 2-week intervals for early, middle, and late treatments starting from the beginning of November; plants in the early treatment had the longest exposure to the outdoor environment. The degree of injury among treated plants was assessed 8 weeks after the start of the experiment. Plant survival was assessed 3 months after the start of the treatment.

To ensure that the outdoor light conditions were the same as much as possible, and to clarify the chilling acclimatization ability including the influence of light, all plants were set so that the light-receiving surface was exposed to abundant sunlight, with the cladode flat sides facing north and south. Plants were watered when the soil was dry. No fertilizer was applied.

Temperature was measured with a high-precision temperature sensor (Ondotori TR-72wf-H, Inc., T&D Corporation, Nagano, Japan). The sensor was placed in a breathable plastic basket 150 cm above ground.

### SOD activity

Cladode chlorenchyma tissues (0.5 g) were collected at 12:00, frozen in liquid nitrogen, and immediately ground with a mortar and pestle. They were ground again with 20 mg of Polyclar AT in 2 ml of grinding medium, which contained 50 mM Tris·HCl (pH 7.5), 5 mM dithiothreitol, 0.2 mM disodium EDTA, 0.5% (w/v) polyvinylpyrrolidone, and 1% (v/v) Triton X-100. The homogenate was centrifuged at 10 000 × *g* for 10 min at 4 °C, and the supernatant was concentrated about tenfold in Amicon Ultra-2 centrifugal filter devices (Merck Millipore Ltd., MA, USA). We used a SOD Assay Kit-WST (Dojindo Co., Kumamoto, Japan), in which the superoxide anion reduces WST-1 ((2-(4-iodophenyl)-3-(4-nitrophenyl)-5-(2 4-disulphophenyl)-2H-tetrazolium, monosodium salt) to yellow formazan, which can be quantified from absorbance at 450 nm. Antioxidants inhibit yellow WST-1 formation. In brief, the supernatants were mixed with WST-1 and then with the enzyme solution and incubated at 37 °C for 20 min. Absorbance at 450 nm was measured in a spectrophotometer. One unit of enzyme activity was defined as the amount of enzyme that caused a 50% decrease in formazan formation. The activity was expressed as units per milligram of protein, and the relative value (%) was based on the value in the control at the start of treatment. Protein contents were determined with the Bradford reagent (Sigma Aldrich Co., St. Louis, MO, USA) according to Bradford (1976). The activity was calculated as the mean of 3 measurements (three plants per treatment).

### Anatomical analysis

Samples for light and transmission electron microscopy were prepared according to Kondo et al. (1998). Small segments of cladode chlorenchyma were fixed immediately in 3% (v/v) glutaraldehyde in 50 mM sodium phosphate (pH 6.8) for 1.5 h, washed in phosphate buffer, post-fixed in 2% OsO_4_ in buffer, dehydrated through an acetone series, and embedded in Spurr’s resin. Ultrathin sections were stained with uranyl acetate and lead citrate, and analyzed at 80 kV acceleration voltage under a transmission electron microscope (TEM, model JEM-1011, JEOL Ltd., Tokyo, Japan). Semithin sections were stained with 1% toluidine blue O.

The thicknesses of the cell wall and cuticle (the cutin and wax layers) were measured using ImageJ software (National Institutes of Health, Bethesda, MD, USA) at 18 points (3 points × 2 sections × 3 plants per treatment) at × 50,000 magnification.

For scanning electron microscopy, epoxy replicas of epidermal cells were prepared according to Green and Linstead (1990). Dental impression material (Extrude Wash, Kerr Corp., Orange, CA, USA) was applied to the cladode surfaces, incubated for 5 min, and peeled off. The negative molds were filled with epoxy resin (2-Ton Epoxy S-31; Devcon Corp., MA, USA), polymerized for 24 h at room temperature, coated with gold and palladium at 10–15 nm thickness, and analyzed at 10–20 kV acceleration voltage under a scanning electron microscope (SEM, model JSM-IT100, JEOL Ltd.).

Cuticular wax coverage (% of cuticular wax per 1.2 mm^2^ surface area of the cladode) was measured using ImageJ software. The procedure included the following steps: (i) cuticular wax was segmented by [Threshold] on the sample image; if segmentation was difficult, a region of cuticle wax was selected with [Polygon selections] and [Cut] to crop it out, leaving a black area; (ii) [Threshold] was adjusted to obtain a black-and-white binarized image; (iii) the area of segmented cuticular wax was analyzed by [Analyze Particles], and cuticular wax coverage was determined as a percentage of the total image area. The coverage was calculated as the mean of 6 measurements (2 sections × 3 plants per treatment) at × 100 magnification.

## Results

### Temperature and antioxidant capacity

**Fig. 2:**
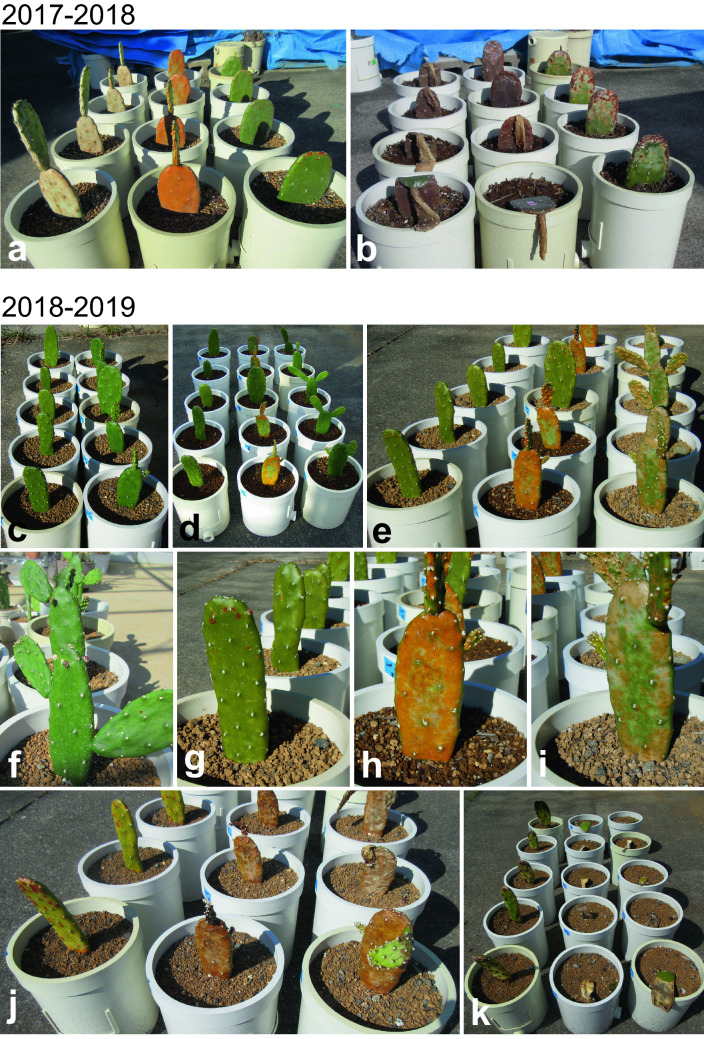
Growth of the cacti outdoors during winter. **a**, **b** 2017–2018. **a** Plants in the early, middle, and late treatments (from right to left) 8 weeks after the start of the study (December 2017). **b** Plants in mid February 2018. **c–k** 2018–2019. Plants in the early, middle, and late treatments (from left to right). **c** Two weeks after the early-treatment plants were placed outdoors, the middle-treatment plants were placed outdoors. **d** Two weeks later, the late-treatment plants were moved outdoors. **e** Eight weeks after the start of the study in December 2018, plants grown in the greenhouse at the same time (control, **f**) and in the **g** early, **h** middle, and **i** late treatments. Plants in **j** late January and **k** early April 2019

To assess the ability of the cacti to acclimatize to chilling, we moved them from indoors to outdoors in November 2017 and investigated their growth. A clear difference in the degree of cladode surface damage was seen after 8 weeks of treatments (Fig. 2a, b). The surface was green in early-treatment plants, orange in middle-treatment plants, and white or discolored in late-treatment plants. Three months after the start of the treatment, early-treatment plants survived, although the cladode margins were damaged, while all other plants died.

**Fig. 3:**
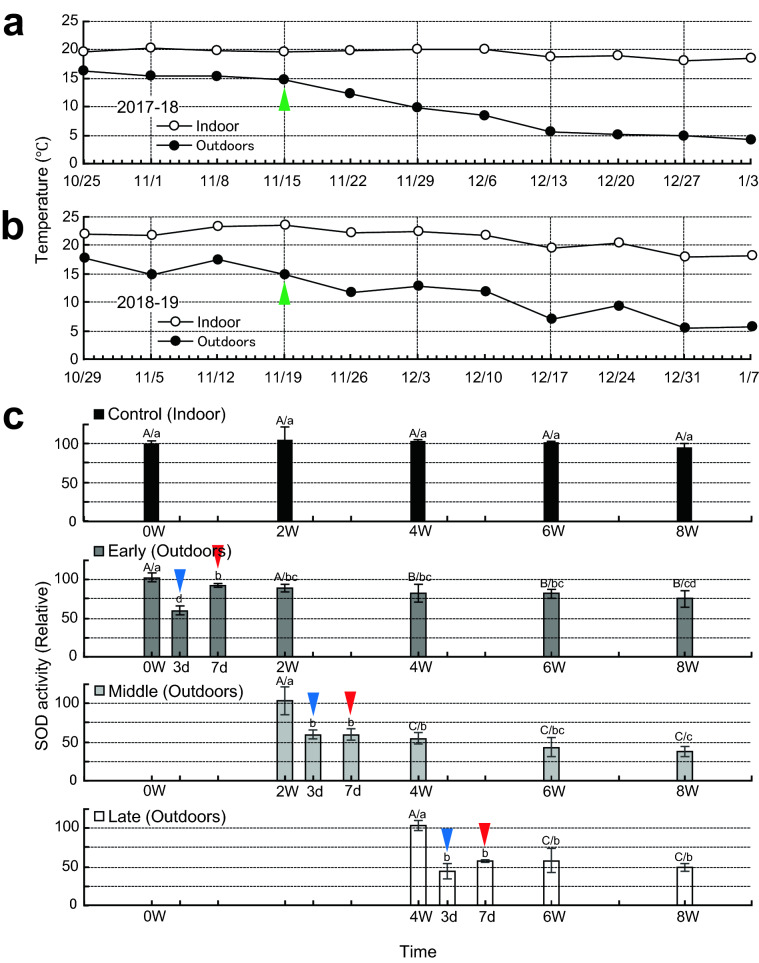
Winter temperature and SOD activity. **a**, **b** Winter temperature in (**a**) 2017–18 and (**b**) 2018–19. Temperature was averaged weekly. **c** SOD activity in 2018–19. The SOD activity values are expressed relative to the indoor value (314 U mg protein^−1^) immediately before the start of the experiment. The first value for each treatment is that of the sample taken just before the shift from indoors to outdoors. A gradual decrease in temperature started when the middle-treatment plants were placed outdoors (green arrowheads). SOD activity decreased to 50–60% after 3 days outdoors (blue arrowheads) in all treatments; it recovered to 90% in the early treatment after 4 days, but did not recover in the middle and late treatments (red arrowheads). Values are means ± SD of 3 plants per treatment. Different capital letters indicate significant difference at *P* < 0.05 between at the same time points. Different lowercase letters indicate significant difference at *P* < 0.05 between time points in the same treatment

The temperature in the greenhouse immediately prior to the outdoor placement of the early-treatment plants was 20.2 °C, and the outdoor temperature was 15.3 °C. The mean temperature during the first 2 weeks outdoors was 15 °C (Fig. 3a). Just around the time when the middle-treatment plants were placed outdoors, the temperature 14.6 °C began to decrease (green arrowhead in Fig. 3a), and the outdoor temperature was mean 11 °C for the first 2 weeks outdoors (Fig. 3a). The late-treatment plants were placed outdoors at 9.7 °C, and the outdoor temperature was mean 7 °C for the first 2 weeks outdoors (Fig. 3a).

In 2018–19, we investigated the relationship between growth and temperature (Fig. 2c–k) as in 2017–18, and measured SOD activity as an indicator of antioxidant capacity. Soon after the middle-treatment plants were placed outdoors, their cladode surface was slightly darker than in the early-treatment plants (Fig. 2c). Soon after the late-treatment plants were placed outdoors, orange-tinged injury was visible in the middle-treatment plants (Fig. 2d). The degree of injury differed among the treatments after 8 weeks of treatment (Fig. 2e). While the indoor-grown control at the same time was green (Fig. 2f), the early-treatment plants were slightly yellowish green (Fig. 2g), the middle-treatment plants were orange (Fig. 2h), and the late-treatment plants had orange lesions at the cladode margins (Fig. 2i). In late February 2019, the middle- and late-treatment plants had the same degree of injury (Fig. 2j) and had fallen (Fig. 2k). In 2018–19, the temperature in the greenhouse immediately prior to the outdoor placement of the early-treatment plants was 21.6 °C, and the outdoor temperature was 14.8 °C. The mean temperature during the first 2 weeks outdoors was 16 °C (Fig. 3b). Just around the time when the middle-treatment plants were placed outdoors, the temperature 14.7 °C began to decrease (green arrowhead in Fig. 3b), and the outdoor temperature was mean 12 °C for the first 2 weeks outdoors (Fig. 3b). The late-treatment plants were placed outdoors at 12.6 °C, and the outdoor temperature was mean 9.3 °C for the first 2 weeks outdoors (Fig. 3b).

Activity in the control (314 U mg protein^−1^; 100%) remained almost constant throughout the treatment period. That in the early-treatment plants decreased to 60% by 3 days after outdoor placement (blue arrowhead in Fig. 3c), but recovered to 93% by 7 days (red arrowhead), and then ranged from 75 to 90%. That in the middle-treatment plants also decreased to 60% after 3 days (blue arrowhead in Fig. 3c), but it remained at 60% without recovery until 7 days (red arrowhead), and then ranged from 38 to 55%. That in the late-treatment plants dropped to 45% after 3 days (blue arrowhead in Fig. 3c), recovered to 58% after 7 days (red arrowhead), and then ranged from 38 to 55%.

### Cell structure in cladode chlorenchyma tissue

**Fig. 4:**
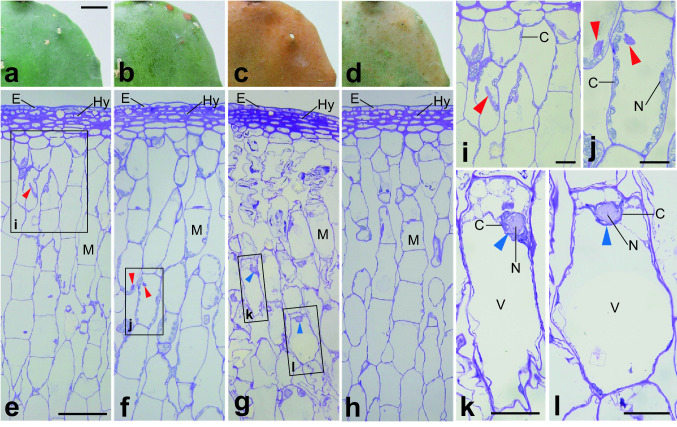
Surfaces and cross-sections of cladodes. **a**, **e** control; **b**, **f** resistant; **c**, **g** sensitive; **d**, **h** semi-sensitive. **i–l** are enlargements of rectangles in **e**, **f**, and **g**. Large organelles distinct from the nucleus (here called unknown organelle-like structures; UKOs) are marked by red arrowheads (**e**, **f**, **i**, **j**). Nuclei clumped by chloroplasts are marked by blue arrowheads (**g**, **k**, **l**). *C* chloroplast, *E* epidermal cell, *Hy* hypodermal cell, *M* mesophyll cell, *N* nucleus, *V* vacuole. Bars: **a** 10 mm; **e** 100 µm; **i–l** 25 µm

**Fig. 5:**
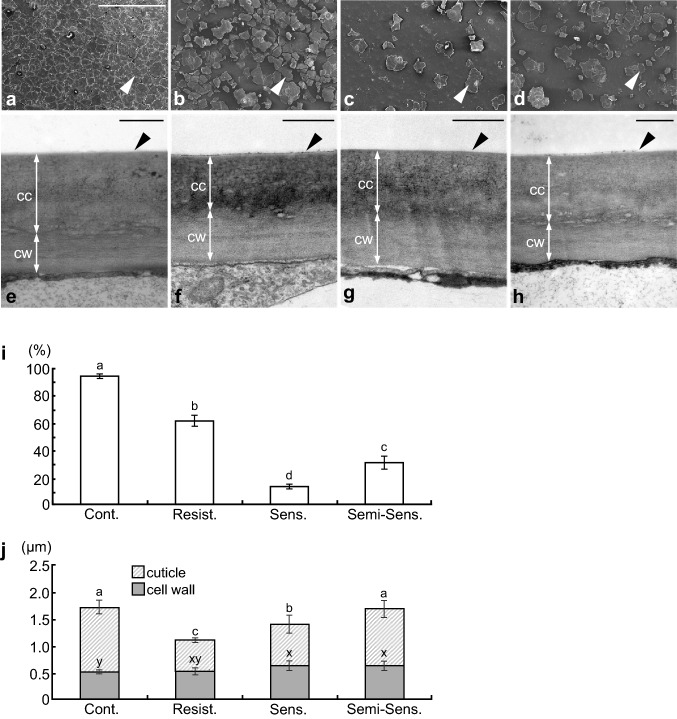
Structure of cladode epidermis. **a**, **e** control; **b**, **f** resistant; **c**, **g** sensitive; **d**, **h** semi-sensitive. **a–d** SEM images of cladode surfaces. **e–h** TEM images of cross-sections of the outer wall of epidermal cells. White arrowheads, puzzle piece–like fragments; black arrowheads, wax layer. *CC* cuticle, *CW* cell wall. Bars: **a** 500 µm; **e–h** 0.5 µm. **i** Cuticular wax coverage (% of cuticular wax per surface area of cladode). Values are means ± SD of 2 sections × 3 plants per treatment. **j** Thicknesses of the cell wall and cuticle (cutin and wax layer). Values are means ± SD of 3 points × 2 sections × 3 plants per treatment. In **i** and **j**, different letters indicate significant difference at *P* < 0.05

**Fig. 6:**
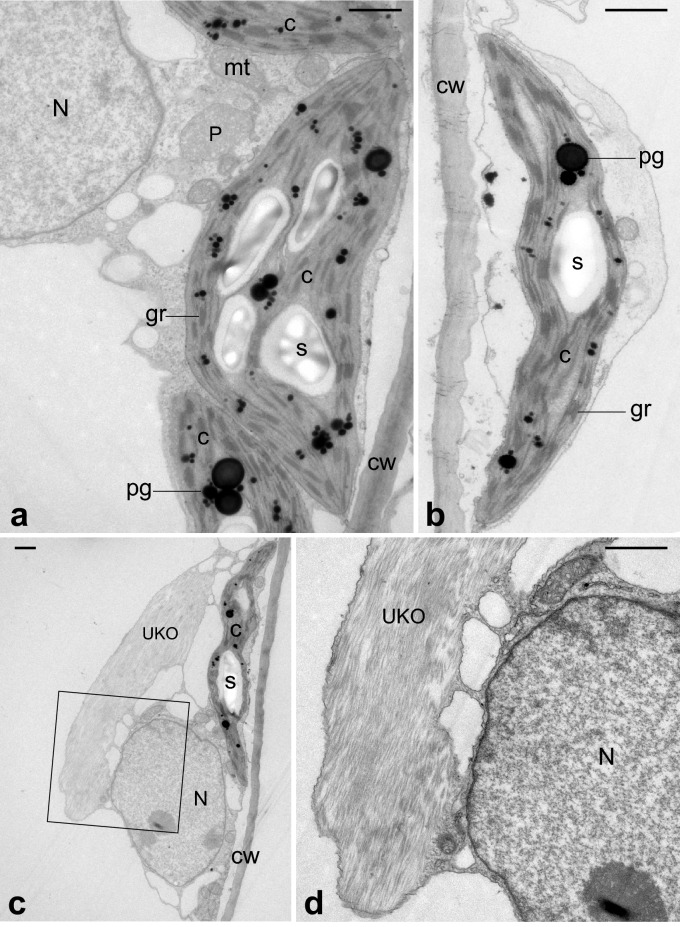
Ultrastructure of mesophyll cells in cladodes of control plants grown in a greenhouse. **a**, **b** shows lens-shaped chloroplasts. **d** is an enlargement of square in (**c**). UKOs (see Fig. 4) are observed near the nucleus and chloroplasts (**c**, **d**). *C* chloroplast, *CW* cell wall, *gr* grana, *mt* mitochondria, *N* nucleus, *P* peroxisome, *pg* plastoglobule, *S* starch granule, *UKO* unknown organelle-like structure. Bars: 1 µm

**Fig. 7:**
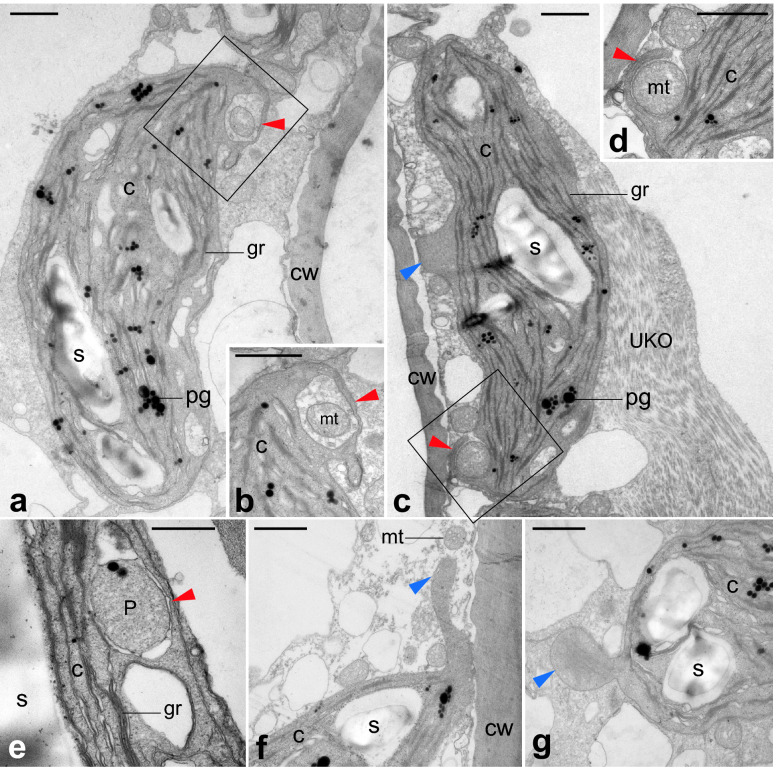
Ultrastructure of mesophyll cells in cladodes of resistant plants surviving low temperature. **b** and **d** are enlargements of rectangles in **a** and **c**, respectively. Chloroplasts incorporate mitochondria (red arrowheads in **a–d**) and peroxisomes (red arrowhead in **e**). Chloroplasts extending protrusions filled with stroma (blue arrowheads in **c**, **f**, **g**). *C* chloroplast, *CW* cell wall, *gr* grana, *mt* mitochondria, *N* nucleus, *P* peroxisome, *pg* plastoglobule, *S* starch granule, *UKO* unknown organelle-like structure. Bars: **a–d**, **f**, **g** 1 µm; **e** 0.4 µm

**Fig. 8:**
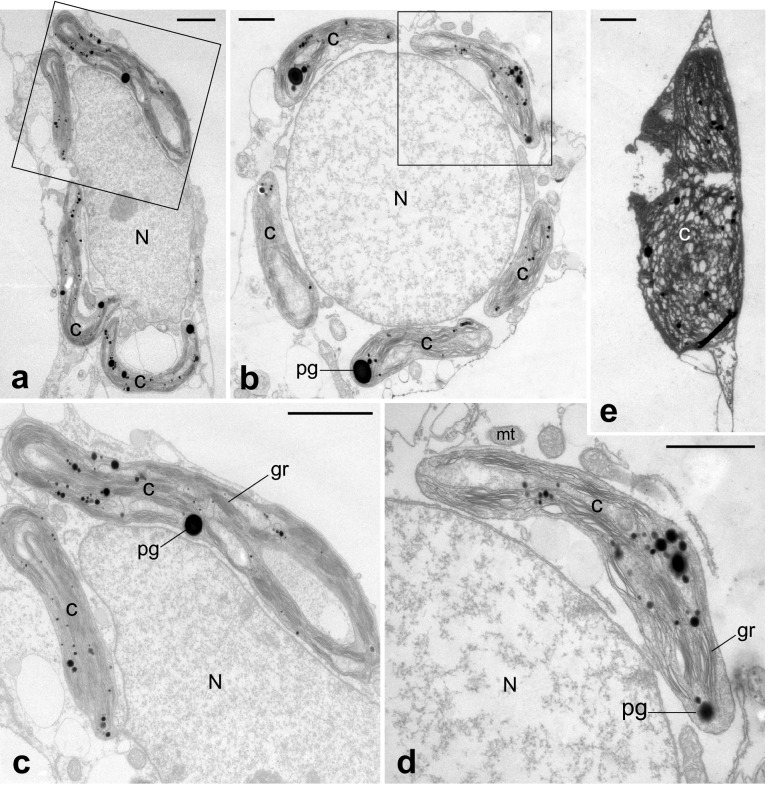
Ultrastructure of mesophyll cells in cladodes of sensitive plants with severe chilling injury. **c** and **d** are enlargements of rectangles in (**a**) and (**b**), respectively. Chloroplasts surround the nucleus (**a–d**). **e** Degraded chloroplast. *C* chloroplast, *gr* grana, *mt* mitochondria, *N* nucleus, *pg* plastoglobule. Bars: 1 µm

**Fig. 9:**
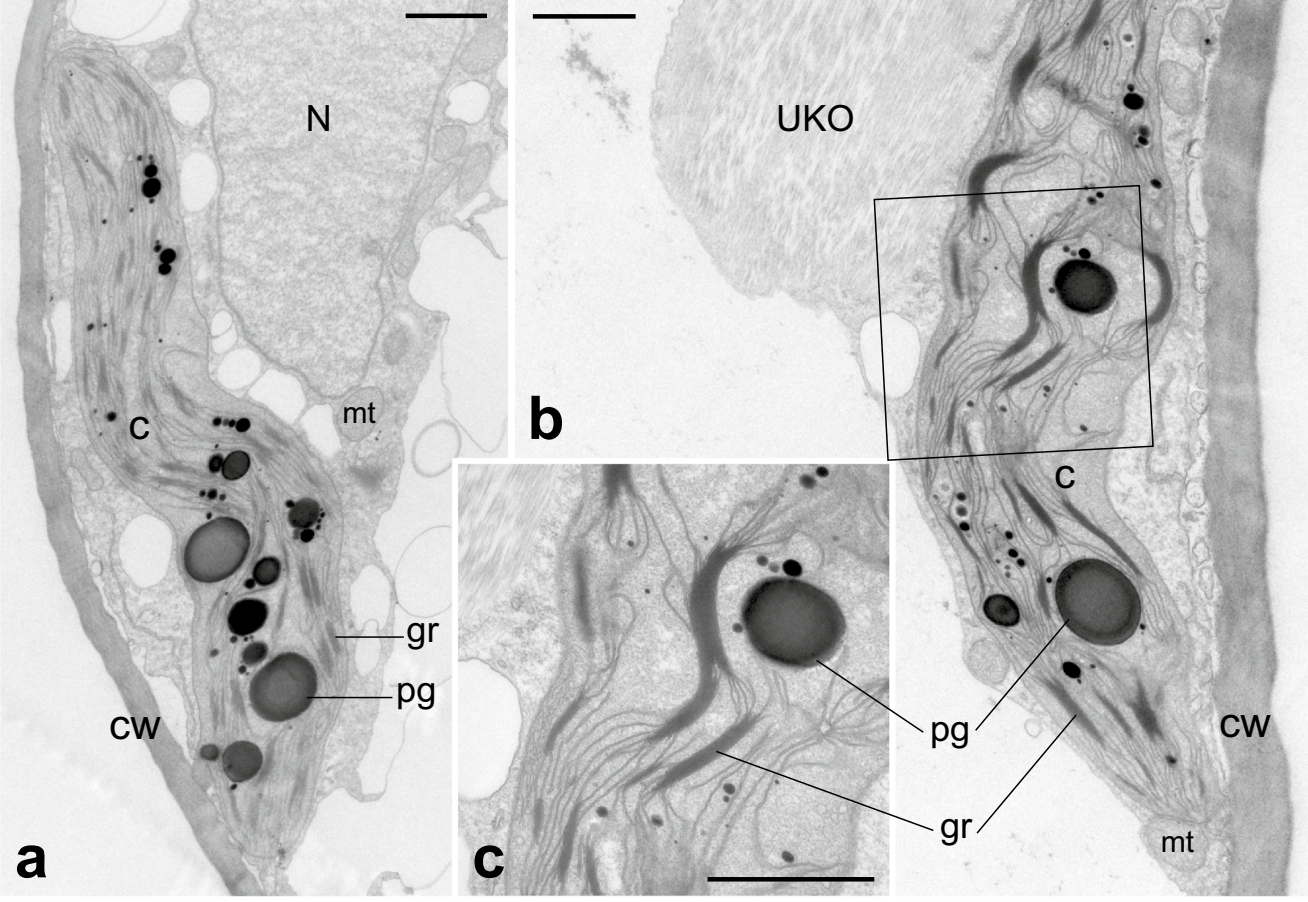
Ultrastructure of mesophyll cells in cladodes of semi-sensitive plants with slight chilling injury. **c** is an enlargement of square in **b**. Large plastoglobules and granules with ruptured thylakoid membranes are characteristic of these plants. *C* chloroplast, *CW* cell wall, *gr* grana, *mt* mitochondria, *N* nucleus, *pg* plastoglobule, *UKO* unknown organelle-like structure. Bars: 1 µm

The analysis was performed 8 weeks after the start of the study. Early-treatment plants eventually overwintered in the low-temperature outdoor environment. On the other hand, middle- and late-treatment plants died without overwintering, but as mentioned above, the late-treatment plants showed less chilling injury than the middle-treatment plants at this same time (Fig. 2h, i). Therefore, in this analysis, in order to clarify the relationship between microstructure and the low-temperature tolerance, the early-, middle-, and late-treatment plants were renamed as resistant, sensitive, and semi-sensitive plants, respectively (Figs. 4, 5, 6, 7, 8 and 9).

The cladode surfaces of cacti shown in Fig. 4a–d correspond respectively to the plants shown in Fig. 2f–i. Four or five layers of thick-walled hypodermal cells were present just beneath the epidermis, and the mesophyll was not differentiated into palisade or spongy tissue (Fig. 4e–h). Under a light microscope, the nucleus and chloroplasts were observed in mesophyll cells, but other large organelles were also present (red arrowheads in Fig. 4e, f, i, j). These organelles were also observed by TEM (see below), and we tentatively named them “unknown organelle-like structures” (UKOs). In the resistant and semi-sensitive plants (Fig. 4f, h), chloroplasts in mesophyll cells were arranged on the lateral side of the cell, as in the control (Fig. 4e), but in the sensitive plants, those immediately below the epidermis were disintegrated (Fig. 4g). The structure of underlying undamaged mesophyll cells differed from that of normal cells (Fig. 4g): a large vacuole occupied the lower part of the cell, and a round, swollen nucleus was localized in the upper part (axial side) as though it were pushed up by the vacuole, with chloroplasts assembled around it (Fig. 4k, l).

The control cladode surface was densely covered with cuticular wax in the shape of puzzle pieces (white arrowheads, Fig. 5a–d). The cuticular wax was 93.4% in control, 60.6% in resistant plants, 12.8% in sensitive plants, and 30.1% in semi-sensitive plants (Fig. 5i). Thus, sensitive plants had the highest cuticular wax detachment, whereas resistant plants had the greatest coverage among the outdoor treatments, five times that of sensitive plants.

TEM images of cross-sections of the outer wall of the cladode epidermal cells showed three typical layers (Shepherd and Wynne Griffiths 2006; Yeats and Rose 2013): cell wall, cutin layer, and cuticular wax layer (Fig. 5e–h). No significant damage to the cell wall or cutin layer was observed in any treatment. Although it is difficult to clearly observe the thin cross-sectional structure of the cuticular wax, the surface of resistant plants looked furrowed, that of sensitive plants looked smooth, and that of semi-sensitive plants appeared to have a raised membrane in comparison with the control (black arrowheads in Fig. 5e–h). This may reflect cuticular wax detachment as observed by SEM (white arrowheads in Fig. 5a–d). The mean thickness of the cell wall was 0.51 µm in the control, 0.54 µm in resistant plants, 0.61 µm in sensitive plants, and 0.62 µm in semi-sensitive plants, with no significant difference among the treatments (Fig. 5j). The mean thickness of the cuticle (cutin and wax layers) was 1.20 µm, 0.58 µm, 0.75 µm, and 1.06 µm, respectively (Fig. 5j), with significant differences among the treatments. The cuticle was thinnest in resistant plants, not in sensitive plants, in which chilling injury was more pronounced on the surface of the cladode. The cuticle thickness of resistant plants was half that of the control plants.

TEM images of mesophyll cells in chlorenchyma tissues are shown in Figs. 6, 7, 8 and 9. In control plants, lens-shaped chloroplasts had a typical arrangement of grana and stroma thylakoids, and contained starch grains and plastoglobules (Fig. 6). In resistant plants, the grana were thinner than in the control, and small rifts in the thylakoid membrane were observed in some places. In many chloroplasts, invaginations incorporated mitochondria and peroxisomes (red arrowheads in Fig. 7) and protrusions filled with stroma were elongated (blue arrowheads in Fig. 7). In sensitive plants, degraded chloroplasts were observed (Fig. 8e). In intact mesophyll cells, chloroplasts were clumped around the nucleus and showed thylakoid swelling and undulation (Fig. 8a–d). In semi-sensitive plants, the chloroplasts were characterized by large plastoglobules and granules with a ruptured thylakoid membrane (Fig. 9).

UKOs were as large and conspicuous as nuclei, but their matrix differed from that of the nucleus, and fibrous traits were observed (Figs. 6, 7, 9).

## Discussion

### Temperature and antioxidant capacity

Here we found that outdoor chilling acclimatization of *N. cochenillifera* can be achieved by exposure to approximately 15 °C for 2 weeks, probably in part owing to antioxidant capacity, which decreased on day 3 after a sudden drop in temperature due to the outdoor placement, but recovered by day 7. Chilling acclimatization was achieved in the early-treatment and plants became resistant to chilling (resistant plants).

In the tropical crop okra (*Abelmoschus esculentus*), storage at 4 °C caused chilling injury, which was attributed to a decrease in SOD activity. 13 °C storage of okra did not cause chilling injury, and SOD activity was 74% higher than that at 4 °C for 15 days. The occurrence of chilling injury at 4 °C is considered to involve the accumulation of superoxide anions due to reduced SOD activity and hydroxyl radicals, the most harmful radicals in the presence of H_2_O_2_ (Phornvillay et al. 2020).

When chilling injury is initiated by the elevation of ROS, the cell acts to reduce the chilling stress, but prolonged chilling periods increase lipid peroxidation, ultimately leading to membrane disruption and disintegration (Lukatkin 2005). In our study, the restoration of antioxidant capacity in the early treatment would prevent serious chilling injury.

Although SOD activity in control cacti (314 U mg protein^−1^) was higher than that in control leaves of Arabidopsis (0.06 U mg^−1^; Yavari et al. 2021), rice (3 U mg^−1^; Poli et al. 2018), and a tree species, *Broussonetia papyrifera* (130 U mg^−1^; Zhang et al. 2013), it was almost equal to that of *Avicennia marina*, a mangrove tree. In the leaves of *A. marina*, SOD activity increases in response to salt and H_2_O_2_, and the latter induces accumulation of SOD mRNA and protein (Fesharaki-Esfahani et al. 2021). *Avicennia marina* can survive under high external salinity, light intensity, and temperature, and heavy metal toxicity (Zeinali et al. 2017), and its SOD characteristics may be important in such tolerance. Further functional characterization of cactus SODs is needed.

### Epidermal structure

In this study, cuticle thickness decreased in plants that were exposed to lower temperatures for longer periods of time; cuticles of the resistant plants were the thinnest. The cuticle consists mainly of cutin and cuticular wax (Shepherd and Wynne Griffiths 2006). Cutin is a polymeric network of oxygenated C_16_ and C_18_ fatty acids cross-linked by ester bonds (Nawrath 2006). Cuticular waxes are complex mixtures of long-chain hydrocarbons, aldehydes, alcohols, acids, and esters derived almost entirely from fatty acids (Kunst and Samuels 2009). In epidermal cells of aerial organs, the bulk of fatty acid synthesis is used for the production of cutin and wax for protection (Bernard and Joubès 2013). Prolonged low temperatures may have reduced cuticular synthesis, since low temperature inhibits photosynthesis and decreases carbohydrate translocation and respiration rate. Cuticular wax was most damaged in sensitive plants, followed by semi-sensitive plants. Thus, resistant plants had the thinnest cuticle but firmly attached cuticular wax. Cuticular wax may be important in the acquisition of low temperature tolerance under strong light.

Cuticle thickness varies from 1 µm to more than 200 µm in species of the *Cactoideae* and *Opuntioideae*, but it lies mostly within 1–10 µm, contrary to the general opinion that the cuticle is thick in most cacti (Loza­Cornejo and Terrazas 2003; Pimienta-Barrios et al. 1993). The difference in cuticle thickness may be related to the ability of the species to conserve water, but no relationship between cuticle thickness and water stress tolerance has been demonstrated in *Opuntia*. In addition, the thickness of the cuticle may act to lower the temperature in the stem, since a thicker cuticle is more reflective of radiation (Terrazas-Salgado and Mauseth 2002).

Perhaps sensitive plants were under increased threat from ROS because of their low antioxidant capacity (Fig. 3c); in addition, the fragility of the epidermis, which allows more light to pass through, must further increase ROS production. The relationship between antioxidant capacity and wax synthesis remains an interesting research challenge.

Rahman et al. (2021) have examined the role of cuticular waxes in reducing low temperature and dehydration stresses in Arabidopsis mutants and transgenic plants with altered cuticular wax formation, and found that cuticular alkane waxes, along with alcohols and fatty acids, promote the avoidance of both ice formation and leaf water loss under dehydration stress. Banana fruit are sensitive to thermal changes, with cold injury at temperatures below 13 °C and green ripening at temperatures above 25 °C (Huang et al. 2021). Changes in surface structure, cuticle chemical composition, and relative expression of cuticle biosynthesis genes in banana fruit during cold storage show that the contents of fatty acids and aldehydes in cuticular wax tend to increase with cold injury (Huang et al. 2021).

Thus, understanding changes in cuticle morphology and chemical composition in response to cold stress will provide useful insights into the initial plant-protective barrier function against the environment. The cuticle composition of cacti should be further investigated.

### Mesophyll cell structure

Chloroplasts in resistant plants formed invaginations that surrounded mitochondria and peroxisomes within a thin layer of chloroplast stroma. These chloroplast pockets have been described in, for example, the mesophyll cells of *Deschampsia antarctica* Desv. growing in Antarctica (Gielwanowska and Szczuka 2005), and tobacco leaves inoculated with tobacco mosaic virus (Shalla 1964). Chloroplast pockets are thought to enhance the association between chloroplasts and other organelles by increasing the contact area, because they are frequently observed under severe conditions (Gielwanowska and Szczuka 2005).

Using serial micrographs obtained under TEM and FIB (focused ion beam) -SEM, Yamane et al. (2018) clearly showed that the rate of chloroplast pocket formation was significantly higher in rice mesophyll cells treated with NaCl than in control cells. In rice leaves under NaCl stress, excess H_2_O_2_ accumulated in chloroplasts was detected at the ultrastructural level by histochemical staining with 3,3-diaminobendizine or cerium chloride (Yamane et al. 2012). These data seem to show a relationship between the formation of chloroplast pockets and high oxidant level.

Clumped chloroplasts were found in intact mesophyll cells of sensitive plants. They have also been found in the mesophyll cells of the succulents *Kalanchoë* and *Zygocactus* under combined light and drought stress (Kondo et al. 2004). In chlorenchyma cells of *Opuntia streptacantha*, Arias-Moreno et al. (2017) found that chloroplasts became densely clumped under salt stress and dispersed upon catalase addition. These findings indicate that H_2_O_2_ is involved in chloroplast clumping.

An unusual aspect of chloroplast clumping in this study is that chloroplasts clustered around the nucleus, although such clumping in *Kalanchoë* has also been reported and analyzed by confocal microscopy (Kondo et al. 2006).

Another interesting point is that many of the clumping chloroplasts were unevenly distributed within mesophyll cells. The lower part of the cell was occupied by large vacuoles, and clumping chloroplasts were localized in the upper part (axial side), as though they were pushed up by the vacuoles. This arrangement of clumping chloroplasts is unusual, since most clumped bodies observed so far are randomly arranged along the intercellular spaces (Kondo et al. 2004). Since decomposed cells and chloroplasts were observed immediately below the hypodermal cells in the same chlorenchyma tissue, the surviving cells must be destroyed and degraded by further exposure to low temperatures.

When chloroplast function is disrupted, the expression of specific nuclear genes changes; this chloroplast-to-nucleus signaling is commonly called “chloroplast retrograde signaling” (Susek et al. 1993). These signal transduction pathways are mediated by the chlorophyll metabolite Mg-protoporphyrin IX, redox balance generated by the photosynthetic electron transport chain, and ROS originating from photochemical reactions. However, it is unclear how these redox signals are relayed to the nucleus (Brunkard and Burch-Smith 2018). Perhaps the chloroplast clumping phenomenon facilitates efficient redox signaling between the nucleus and chloroplasts under severe stresses such as drought, cold, and salt.

It is not clear what factors were involved in the dramatic changes in the mesophyll cells, but as mentioned above, ROS, especially H_2_O_2_, are probably involved and their effects may depend on their threshold levels. Chloroplast pockets may contribute to reducing oxidative stresses, because they were observed only in resistant plants, in which antioxidant capacity was higher than in sensitive plants, and the pockets most frequently contained mitochondria, which are the major sites of ROS production (Foyer and Noctor 2003). For low temperature acclimatization, maintaining high antioxidant capacity is important, and the chloroplast pockets may help. Once the cell reaches the limit of its capacity to tolerate ROS, it may turn to the last bastion of defense, chloroplast clumping, and further excess ROS may lead to cell degradation.

Fisher et al. (2022) have demonstrated that singlet oxygen, a ROS, can trigger chloroplast swelling, changes in chloroplast internal structure, and chloroplast interactions with the central vacuole before degradation. Damaged chloroplasts are targeted for degradation; chloroplast protrusion into the vacuole interior and vesicle formation known as “blebbing” may be involved in it. Although Fisher et al. (2022) found no specific arrangement of chloroplasts, these structural changes may be one of the mechanisms by which cells target damaged or senescing chloroplasts for turnover.

A chloroplast aggregate body has been found in the center of the mesophyll cells of a C_4_ plants, *Bienertia cycloptera* (Chenopodiaceae) in which C_4_ photosynthesis occurs within a single cell (Voznesenskaya et al. 2001). This cell type is unique in terms of the general arrangement of chloroplasts; the functional division within it has been clarified by the analysis of enzyme localization (Voznesenskaya et al. 2002). In the cells of *N. cochenillifera*, the localization analysis of enzymes involved in photosynthesis and antioxidants may reveal some unique functions.

The semi-sensitive and sensitive plants eventually died in winter, but whether the chloroplast structure of the semi-sensitive plants observed here changed to the structure of the sensitive plants needs to be further investigated. Since the treatment of the semi-sensitive plants began at the greatest temperature difference between outdoors and indoors, this sudden drop in temperature may have led to specific changes in the semi-sensitive plants that differ from the sensitive plants.

It remains to be seen what kind of cell organelle the UKO found in this study is. The UKO may be nothing more than an accumulation of some substance, rather than an organelle. The UKO appears to contain fibers. Freeman (1973) morphologically examined the plastids from the development of seedlings of *Opuntia basilaris*. He found fibrillar inclusions in the plastids at all developmental stages of the epidermis and mesophyll cells of *O*. *basilaris* leaves. In addition, fibrillar inclusions were also found in the etioplasts of *O*. *basilaris* leaves grown in the dark, suggesting that these structures are not generated as a direct result of photosynthesis. The UKO does not have the characteristic lamellar structure of the plastids, but is occupied only by fibrous ones. Therefore, it is inferred that the UKO is distinct from the plastids. The UKOs were found in all of the treatments in this experiment, suggesting that they are originally present in the mesophyll cells of mature cladodes. We will investigate this in detail during plant growth in this and other cactus species.

## Data Availability

No data available.
